# Editorial: Biomechanics in orthopaedic diseases and surgery, volume II

**DOI:** 10.3389/fbioe.2025.1768760

**Published:** 2026-01-27

**Authors:** Cheng-Kung Cheng, Yih-kuen Jan, Jingwei Zhang

**Affiliations:** 1 School of Biomedical Engineering and Engineering Research Center for Digital Medicine of the Ministry of Education, Shanghai Jiao Tong University, Shanghai, China; 2 Rehabilitation Engineering Lab, Department of Health and Kinesiology, University of Illinois at Urbana Champaign, Champaign, IL, United States; 3 Department of Orthopaedics, Ninth People’s Hospital, Shanghai Jiao Tong University School of Medicine, Shanghai, China

**Keywords:** biomechanics, fixation, orthopaedic diseases, surgery, trauma

## Introduction

Biomechanics serves as the indispensable bridge between mechanical engineering principles and clinical orthopaedics, offering quantitative insights that drive surgical innovation and implant design. In this Research Topic, “Biomechanics in Orthopaedic Diseases and Surgery, Volume II,” we present 16 studies that collectively advance our understanding of musculoskeletal pathology and treatment. By leveraging diverse methodologies-including finite element analysis (FEA), prospective gait analysis, retrospective clinical cohorts, and molecular mechanobiology—these contributions address critical challenges ranging from fracture fixation stability to the microscopic determinants of bone regeneration.

## Optimizing fixation and reconstruction in the hip and femur

The proximal femur remains a focal point of biomechanical research due to the high prevalence of fractures and the complexity of reconstruction. Several studies in this volume utilized computational modeling to refine fixation strategies for femoral neck fractures.

Zhang et al. used an *in silico* model to evaluate the Femoral Neck System (FNS) for treating Pauwels type III fractures. The authors found that the Maximum Interfragmentary Gap (MIG) had the highest weight in stability scoring, and, crucially, rotating the bolt 5° anteriorly relative to the femoral neck axis yielded the most stable construct. Addressing material biocompatibility, Cui et al. explored the design of magnesium alloy bionic cannulated screws (MABCS). By simulating fracture-healing stages, the study determined that a 10% screw diameter for bionic holes provides the optimal balance between promoting biphasic cortical-cancellous healing and maintaining mechanical integrity.

Beyond standard fractures, this volume also tackles complex reconstructive scenarios. For the challenging “ultrashort” residual proximal femur (URPF) following tumor resection, Hou et al. introduced a novel triangular fixation stem (TFS). Biomechanical testing on Sawbones models demonstrated that the TFS significantly reduced axial displacement and increased torque resistance compared to conventional stems, offering a promising solution for revision and oncology patients. In the field of hip preservation, Sun et al. investigated the biomechanical predictors of success in Surgical Hip Dislocation combined with Femoral Neck Rotational Osteotomy (SHD-FNRO) for osteonecrosis. Both clinical follow-up and FEA confirmed that the postoperative Neck-Shaft Angle (NSA) is statistically associated with hip preservation outcomes, emphasizing the need for precise osteotomy planning. Complementing these macroscopic studies, Feng et al. provided a micro-structural analysis of the proximal femur. By digitally simulating osteoporosis, the authors revealed that the femoral neck is significantly more susceptible to structural deterioration than the femoral head, with a 5% mass loss resulting in a disproportionate 12% reduction in mechanical performance.

## Refining knee surgery: from trauma to arthroplasty

Innovations in knee surgery presented in this volume span the spectrum of trauma, realignment, and replacement, with a strong emphasis on preserving native biomechanics. In patellar fracture management, Gao et al. combined FEA with a clinical study of 66 patients to validate the “dual tension band” technique. The results were compelling: patients treated with this novel technique exhibited higher Insall-Salvati Indices (0.93 vs. 0.85) and greater range of motion, thereby reducing the incidence of postoperative patella baja compared with traditional single-band fixation. To improve the fidelity of finite element models for tibial plateau fractures, Besa et al. quantified the regional bone density heterogeneity of the tibia. The study established that the subchondral and medial bone regions have significantly higher density than their metaphyseal and lateral counterparts, a nuance that future simulations must incorporate to improve accuracy.

In the context of elective knee surgery, Li et al. addressed the debate regarding changes in posterior tibial slope (PTS) during High Tibial Osteotomy (HTO). The researchers found that the axial plane inclination of the hinge axis is a critical determinant of PTS; specifically, as the inclination angle increases, PTS increases, necessitating precise surgical adjustments to the opening gap ratio. For unicompartmental knee replacement (OUKR), Min et al. investigated the mechanical causes of tibial periprosthetic fractures. Contrary to conventional wisdom, the FEA results suggested that in small patients with severe varus anatomy, the tibial component should be placed in approximately 5° of varus to minimize stress concentrations and avoid fracture. Looking beyond the operated joint, Wang et al. utilized gait analysis to explore the systemic effects of unilateral Total Knee Arthroplasty (TKA). The study revealed significant kinematic improvements—such as enhanced step length and vertical translation—in the untreated contralateral knee, suggesting that TKA facilitates a bilateral biomechanical recovery.

## Advanced solutions for the spine and upper extremity

Surgical interventions in the spine and upper limbs often require balancing decompression with stability. Lian et al. compared three surgical approaches for cervical ossification of the posterior longitudinal ligament (OPLL). The finite element results indicated that Anterior Controllable Antedisplacement and Fusion (ACAF) and X-shape corpectomy (ACXF) provided superior stability and lower risk of cage subsidence compared to standard Anterior Cervical Corpectomy and Fusion (ACCF). In the lumbar spine, Li et al. quantified the limits of safety for facet joint resection during percutaneous endoscopic transforaminal discectomy (PETD). The study warned that resecting more than 50% of the ventral superior articular process leads to a significant increase in stress on the L3-4 segment, potentially causing iatrogenic instability. In the upper extremity, Yin et al. focused on the Sauvé-Kapandji procedure for the wrist. Through FEA, the authors demonstrated that reconstructing the Distal Oblique Bundle (DOB) is essential for stabilizing the proximal ulnar stump, significantly reducing stress peaks and displacement during pronation and supination. For functional restoration in tetraplegia, Smith et al. employed musculoskeletal simulation to optimize tendon transfers for lateral pinch grasp. The findings challenge the standard of care, suggesting that transferring clusters of multiple muscles generates better coordination and less joint hyperflexion than the single flexor pollicis longus transfer.

## Mechanobiological insights into tissue health finally

This Research Topic bridges the gap between mechanical function and biological composition. Blázquez-Carmona et al. investigated the healing of woven bone, using Raman spectroscopy and nanoindentation to establish power laws for predicting elastic modulus. The study concluded that microarchitecture plays a more dominant role than chemical composition in determining tissue stiffness during regeneration. Addressing muscle pathology, Liu et al. explored the mechanisms of sarcopenia. The researchers discovered that excessive serum diamine oxidase (DAO) acts as a metabolic disruptor that inhibits myoblast migration and fusion via the Fbln1/FAK pathway, offering a novel metabolic-biomechanical target for treating age-related muscle decline.

## Conclusion

The 16 articles compiled in Volume II of “Biomechanics in Orthopaedic Diseases and Surgery” demonstrate the transformative power of multidisciplinary research. From optimizing screw threads and osteotomy angles to decoding the molecular signals of muscle weakness, these findings provide concrete evidence to refine clinical practice. We hope this Research Topic inspires further integration of biomechanical analysis into routine surgical planning and therapeutic development.

